# The Influence of Brightness on Functional Assessment by mfERG: A Study on Scaffolds Used in Retinal Cell Transplantation in Pigs

**DOI:** 10.1155/2012/263264

**Published:** 2012-02-22

**Authors:** A. T. Christiansen, J. F. Kiilgaard, M. Smith, R. Ejstrup, G. E. Wnek, J. U. Prause, M. J. Young, H. Klassen, H. Kaplan, M. La Cour

**Affiliations:** ^1^Department of Ophthalmology, Glostrup Hospital, Copenhagen University Hospital, Nordre Ringvej 57, 2600 Glostrup, Denmark; ^2^NuVention Solutions Inc., Valley View, OH 44125, USA; ^3^Case Western Reserve University, Cleveland, OH 44106, USA; ^4^Eye Pathology Institute, University of Copenhagen, 2100 Copenhagen, Denmark; ^5^Schepens Eye Research Institute, Department of Ophthalmology, Harvard Medical School, Boston, MA 02114, USA; ^6^The Gavin Herbert Eye Institute and Stem Cell Research Center, University of California, Irvine, CA 92697, USA; ^7^Department of Ophthalmology and Visual Sciences, Kentucky Lions Eye Center, University of Louisville, Louisville, KY 40208, USA

## Abstract

To determine the effect of membrane brightness on multifocal electroretinograms (mfERGs), we implanted poly lactic-co-glycolic acid (PLGA) membranes in the subretinal space of 11 porcine eyes. We compared membranes with their native shiny white color with membranes that were stained with a blue dye (Brilliant Blue). Histological and electrophysiological evaluation of the overlying retina was carried out 6 weeks after implantation. Histologically, both white and blue membranes degraded in a spongiform manner leaving a disrupted outer retina with no preserved photoreceptor segments. Multifocal ERG revealed the white membranes to have a significantly higher P1-amplitude ratio than the blue (*P* = 0.027), and a correlation between brightness ratio and P1-amplitude ratio was found (*r* = 0.762). Based on our findings, we conclude that bright subretinal objects can produce normal mfERG amplitude ratios even when the adjacent photoreceptors are missing. Functional assessment with mfERG in scaffold implant studies should therefore be evaluated with care.

## 1. Introduction

Subretinal transplantation of stem cell-like cells, such as retinal progenitor cells (RPCs), has shown great restorative potential in a number of animal models of retinal degeneration [1]. Although these cells retain the ability to migrate to the outer retina, differentiate to mature photoreceptors, and generate synapses with existing cells [1], the number and the organization of surviving cells fall short of that needed to restore useful vision.

The use of scaffolds in subretinal transplantation has been shown to increase the number of delivered and surviving cells, to enable a more precise and localized delivery [2] and to promote differentiation and organization of grafted RPCs [2–4]. Furthermore, scaffolds can be loaded with regulatory and modulating drugs to further assist differentiation, function, and survival [5, 6].

As scaffold material, poly lactic-co-glycolic acid (PLGA) has shown good results for subretinal transplantation of RPC in mice [2, 5], rats [4], and pigs [3] and is among the more commonly used materials for generating scaffolds [7].

We wanted to use multifocal electroretinography (mfERG) to assess the functional impact of implantation of scaffolds made of PLGA and other polymers. To our surprise, we found that the mfERG amplitudes derived from retina apparently overlying the PLGA membranes were normal despite histologically verified destruction of the outer retina in the same area. We then hypothesized that the brightness of the white transplanted membranes caused the mfERG stimulus to be backscattered and so produced a stray light-induced response. To test this hypothesis, we first implanted a batch of PLGA membranes stained with a blue dye, and therefore with reduced surface brightness. Hereafter, we compared the mfERG amplitude ratios derived from areas overlying white PLGA membranes with those derived from areas overlying blue PLGA membranes and furthermore correlated the mfERG amplitude ratios with the surface brightness ratios over the transplanted PLGA membranes.

## 2. Material and Methods

### 2.1. Animals

 All experiments were performed in compliance with The Association for Research in Vision and Ophthalmology (ARVO) Statement for the Use of Animals in Ophthalmic and Vision Research. The Danish Animal Experiments Inspectorate granted permission for the use of the animals (permission 2007/561-768). Trained veterinary nurses and technicians carried out all handling of the animals. 

A total of 21 female domestic pigs of Danish Landrace/Duroc/Hampshire/Yorkshire breed were used for these experiments (age 3-4 months; weight 23–30 kg). Only left eyes underwent membrane transplantation. The animals were premedicated with Tiletamine 1.19 mg/kg, Zolazepam 1.19 mg/kg (Zoletil 50 Vet Virbac SA, Carros, France), Methadone 0.24 mg/kg (Nycomed, Roskilde, Denmark), Ketamine 1.43 mg/kg (Intervet, Skovlunde, Denmark), and Xylazine 1.24 mg/kg (Intervet, Skovlunde, Denmark). Thereafter, anesthesia was maintained with continuous intravenous infusion (i.v.) of propofol 15 mg/kg/h (Fresenius Kabi, Bad Homburg, Germany). The animals were endotracheally intubated and artificially ventilated on 34% O_2_. During anesthesia, the animals were placed resting on their elbows to minimizing the impact on the cardiovascular system [8]. In order to avoid hypothermia, the animals were wrapped in a blanket during anesthesia.

### 2.2. Surgical Procedure

 Eyes were anesthetized, dilated, and disinfected, and a standard three-port core vitrectomy was performed as previously described [9]. In brief, the infusion line was secured inferiorly (Ringer Lactate; SAD, Copenhagen, Denmark), and the vitreous was removed during endoillumination using a 20 gauge (G) vitrector (Karl Storz GmbH, Tuttlingen, Germany). The posterior hyaloid was meticulous removed in the visual streak and optic disc area. A subretinal bleb in the visual streak area was raised by injection of Ringer Lactate (SAD, Copenhagen, Denmark) through a 41 G cannula (ref. 1270; DORC International BV, Zuidland, the Netherlands). To gain access to the subretinal space, a retinotomy was performed in the temporal aspect of the bleb using endodiathermy (Storz Premiere, Bausch and Lomb; energy set 15%, output range 7.5 Watts nominal at 100 ohms) and automated scissors (Storz Premiere, automatic scissors). This allowed a large piece of membrane (approx. 12 mm^2^) to be inserted in the visual streak area. DORC's combined spatula/peeling forceps (Ref. 1297, DORC, Netherlands) were used for this process. In order to secure the membrane, a partial fluid-air-exchange with drainage at the retinotomy site was performed after the membrane was placed subretinally. Sclera and conjunctiva were sutured with 7–0 coated vicryl (Ethicon, Norderstedt, Germany). After the procedure, intraocular pressure was evaluated with bimanual palpation, and indirect ophthalmoscopy was performed to ensure correct placement of the membrane and absence of bleeding and retinal detachment. Finally, topical application of chloramphenicol ointment was given, and the eye was patched (Kloramfenikol “DAK”; Nycomed, Roskilde, Denmark). In order to ensure reliable mfERG recordings, animals with any surgical complication, such as bleeding, surgical lens damage or retinal detachment as well as animals with significant opacities in the media were excluded from the study.

### 2.3. Follow-Up Procedure

Six weeks after-surgery, animals were reanesthetized as previously described [8] with addition of a neuromuscular blocker to avoid eye movement, 2 mg/h i.v. Pancurium Bromide (Oss, Organon, Holland). Infrared (IR) fundus imaging with an external IR light source was used as previously described [8]. Color fundus photos obtained with a Zeiss fundus camera just prior to euthanasia (Zeiss FF450 plus-IR). Multifocal ERG was recorded on both eyes. Recordings were conducted in an electrically shielded room under standardized lighting conditions, and dilated eyes were adapted to room light for 15 minutes. A Burian-Allen bipolar contact lens electrode (VERIS Infrared Illuminating Electrode, EDI, Inc., Red Wood, CA) was placed on the cornea with a gel (Viscotears, Novartis, Copenhagen, Denmark) as contact fluid. A reference electrode was placed behind the right ear, and the animal was electrically grounded. To minimize the effect of anesthesia on the mfERG recording, the left membrane-implanted eye was always recorded within the first two hours of anesthesia [8], and the two eyes were recorded within a timeframe of 30 min.

At completion of follow-up studies, animals were euthanized by a lethal injection of 20 mL pentobarbital 200 mg/mL (Royal Veterinary and Agricultural University, Copenhagen, Denmark). Eyes were then enucleated and prepared for histology as previously described [8]. 

### 2.4. mfERG Settings

Recordings were obtained using VERIS Science 5.0.1 with visual stimulus displayed on a 1.5 inch cathode ray tube monitor integrated in the stimulus-camera (EDI Inc., Redwood City, CA, USA). A stimulus pattern of 241 unscaled white (200 cd/m^2^) and black (2 cd/m^2^) hexagons with a frame rate of 75 Hz and 16 samples per frames was used to obtain the best spatial resolution with a reasonable signal-to-noise ratio [8]. The m-sequence exponent was 15 and the durations of recordings were 7.17 minutes. The signals were band-pass filtered outside 10–300 Hz. There was no spatial averaging and only 1st order kernels were used.

### 2.5. mfERG Analysis

**Figure 1:**
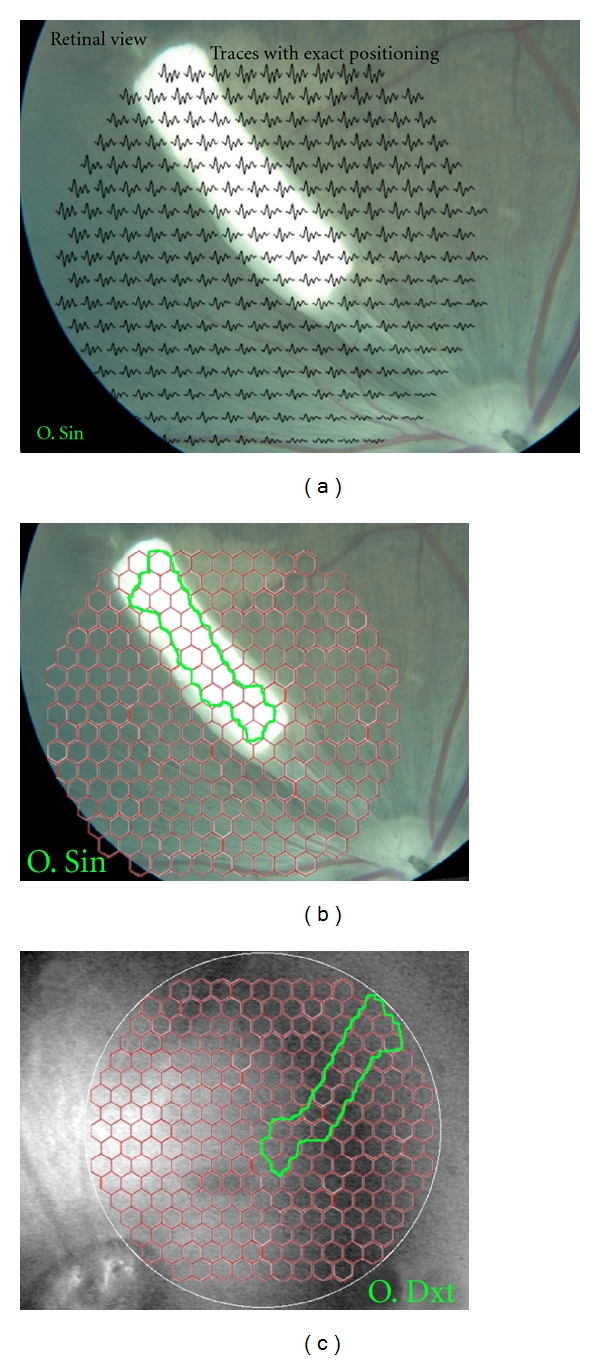
Fundus photos of both eyes 6 weeks after white PLGA transplantation. (a) Color fundus photo aligned with multifocal electroretinographic (mfERG) traces of the 241 unscaled stimulated hexagons. The bright reflective properties of the membranes are clearly visible. (b) Color fundus photo of the left membrane-transplanted eye aligned with hexagon grid used for mfERG recording. Area included as membrane is marked with a green line. (c) Infrared fundus photo of fellow right untouched eye with hexagon grid used for mfERG recording. The green line marks area included at membrane corresponding area for calculation of P1-amplitude ratio.

**Figure 2:**
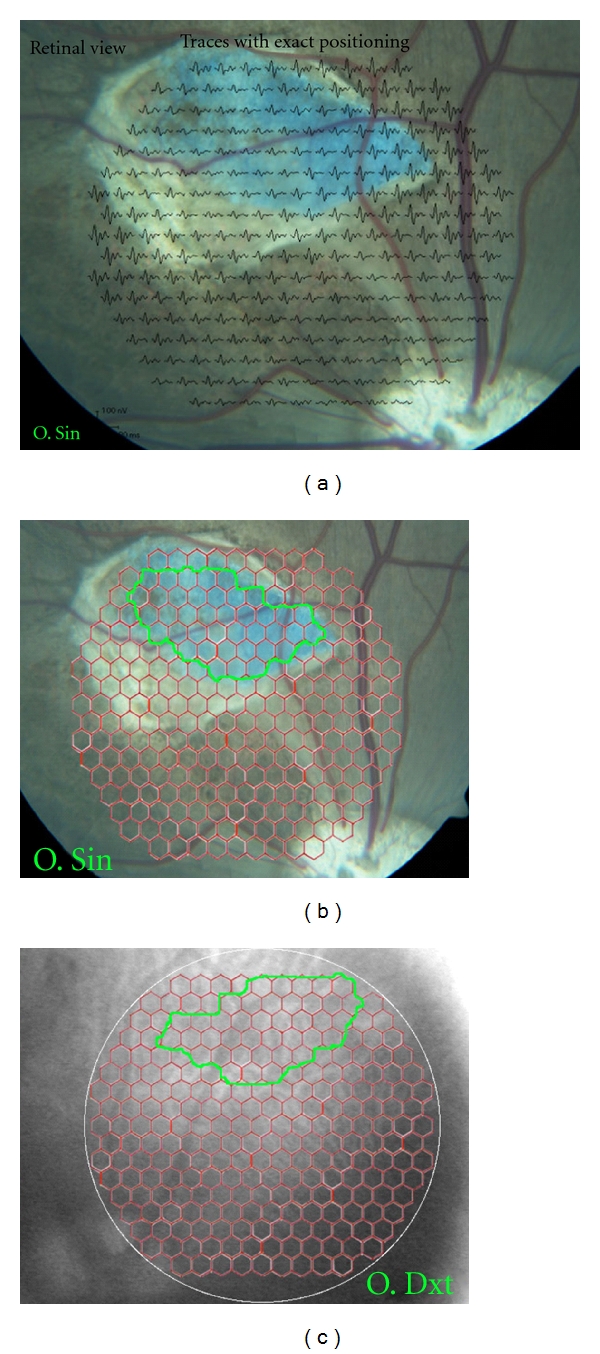
Fundus photos of both eyes 6 weeks after blue PLGA transplantation. (a) Color fundus photo aligned with multifocal electroretinographic (mfERG) traces of the 241 unscaled stimulated hexagons. The membrane is surrounded by choroidal neovascularization but does not appear to reflect light in the manner of the white PLGA. (b) Color fundus photo of the left membrane-transplanted eye aligned with hexagon grid used for mfERG recording. Area included as membrane is marked with a green line. (c) Infrared fundus photo of fellow right untouched eye with hexagon grid used for mfERG recording. The green line marks area included at membrane corresponding area for calculation of P1-amplitude ratio.

To identify hexagons representing retina with an underlying membrane, an alignment of the IR fundus photo of the left eye with the stimulus grid from the VERIS system and the corresponding Zeiss color fundus photo was performed using Photoshop (version 10.0, Adobe Systems Inc., San Jose, CA, USA). This was done for every individual left (experimental) eye in the study, and the hexagons completely within the membrane-affected area were labeled. The corresponding area in the right (control) eye was delineated from the VERIS IR fundus photo (Figures 1(b), 1(c), 2(b), 2(c)). The averages of the P1-amplitudes derived from hexagons within the membrane-affected area were calculated. The averages of P1-amplitudes in the right eye derived from the corresponding retinal area were also calculated as well as the ratios between these P1 amplitudes from the two eyes. Independent-samples *t*-test was used to test for equality of means between the two membrane color ratios (SPSS Statistics, version 17.0). 

To control the comparability of the right and left eye, the visual streak was localized using the 3D multiplot of the Veris program, and mfERG for the entire visual streak was recorded and compared.

### 2.6. Brightness Analysis

Color fundus photos were used to evaluate the brightness of the subretinally transplanted membranes. Area of interest was marked and measured in Photoshop on a scale ranging from 0 (black point) to 255 (white point) as described by Hubbard et al. [10]. The ratio between the membrane area and the optic disc brightness was used to even out differences in the fundus photo flash intensity. The difference in brightness ratios between the two membrane colors and Pearsons correlation between brightness ratio and mfERG P1-amplitude ratio were calculated using SPSS (SPSS Statistics, version 17.0). Ratios were plotted in Figure 5 using SigmaPlot (SigmaPlot for Windows 11.0, Systat Software Inc., CA, USA).

### 2.7. Histology

The part of the formaldehyde (4%) fixated eye containing the PLGA membrane was cut out and embedded in paraffin. Sections of 5 micrometer through the membrane were then stained with haematoxylin and eosin (HE) and evaluated by light microscopy.

### 2.8. Membranes

White (undyed) membranes were constructed by transferring a solution of 15 weight percent (wt%) PLGA in CHCl3 to a 5 mL syringe attached to a blunt tipped 18 G stainless steel needle. Hereafter, electrospinning was carried out through the application of a 15 kV positive voltage to the polymer solution. The solution was then fed via a syringe pump at a constant mass flow rate of 1 mL/h. Fibers were collected on a stainless steel grounded rotating drum until a nonwoven mat was formed. For the blue PLGA membranes, 2 wt% of Brilliant Blue FCF was added to the 15 wt% PLGA solution.

## 3. Results

A total of 21 pigs underwent PLGA-membrane transplantation surgery, whereof 12 had white membranes transplanted. Ten pigs, 4 with blue and 6 with white membranes, were excluded due to postoperative complications shown in Table 1. Included in the study were 11 pigs, 6 with white membranes, and 5 with blue membranes. The high modulus of the used PLGA membranes eased the insertion into the subretinal space. Contrarily the lack of compliance complicated the precise delivery within the bleb.

It was possible to obtain good mfERGs with acceptable signal-to-noise ratios in both the left and right eye in all included pigs. Evaluation of the recorded mfERGs shows that the visual streak of the fellow eyes is comparable. Further the ratios reveal a tendency for the right visual streak to produce a lower mfERG signal than the fellow left visual streak (Table 2). Multifocal ERG of the membrane and the membrane-corresponding areas revealed a significant difference between mean P1-amplitude ratios from white and blue membrane areas (*P* = 0.027, Table 3).

**Figure 3:**
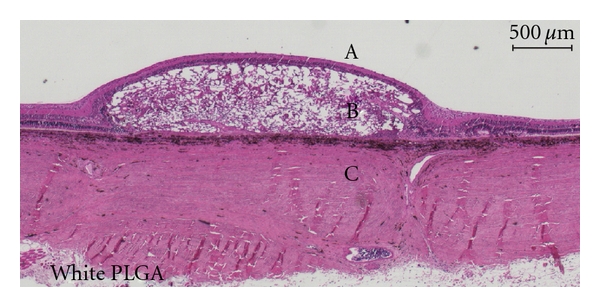
Micrograph of Hematoxylin- and Eosin-stained porcine retina after subretinally transplanting white PLGA membrane (B). The membrane is degrading in a spongiform manner disrupting and partly destroying the outer retina with no preserved photoreceptor segments and a flattened, disrupted, or completely missing outer nuclear layer. The inner nuclear layer is relatively intact and there is no sign of severe inflammation in either retina or choroid. Retina adjacent to the membrane appears intact. Vitreous body is indicated by “A” and sclera by “C”.

**Figure 4:**
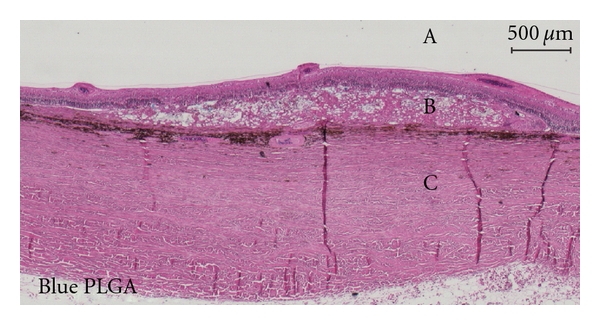
Micrograph of Hematoxylin- and Eosin-stained porcine retina after subretinally transplanting blue PLGA membrane (B). The membrane is degrading in a spongiform manner disrupting and partly destroying the outer retina. The disruption seems to vary, but no photoreceptor segments are preserved. The outer nuclear layer is relatively well preserved in a few places but generally flattened and disorganized or missing. The inner nuclear layer is relatively intact. No sign of severe inflammation in retina or choroid. Retina adjacent to the membrane appears intact. Vitreous body is indicated by “A” and sclera by “C”.

Histological examination showed, however, no major differences between blue and white membranes. Both membranes were associated with spongiform degeneration of the overlying retina, together with a giant cell foreign body reaction. No severe inflammation was seen in either retina or choroid. The retina adjacent to the membrane was intact but the overlying retina had complete loss of photoreceptor outer and inner segments. The outer nuclear layer (ONL) was marginally more preserved over the blue PLGAs but was generally either flattened, disorganized, or completely missing whereas the inner nuclear layer (INL) seemed relatively intact over both white and blue membranes (Figures 3 and 4).

 In spite of the similar outer retinal destruction seen histologically (Figures 3 and 4), mfERG traces over the membranes differed between the two colors with near-normal P1-amplitudes over the white membranes and reduced P1-amplitudes over the blue membranes (Figures 1(a) and 2(a), Table 3).

**Figure 5:**
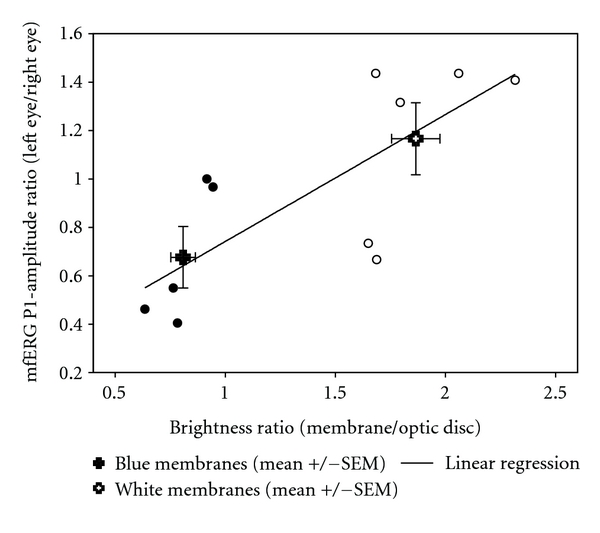
Mean values of white and blue membranes and correlation between brightness ratio (membrane/optic disc) and P1-amplitude ratio (left/right). Large symbols represent mean ± SEM for white and blue membranes and show a significant difference in P1-amplitude ratio (*P* = 0.027) and brightness ratio (*P* < 0.001). When observed together, the blue (●) and white (°) membranes show a significant correlation (*r* = 0.  762; *P* = 0.006).

Brightness ratios from all included animals were obtained and showed a significant difference between white and blue membranes (Figure 5). A correlation between brightness ratio and P1-amplitude ratio could be demonstrated (*r* = 0. 762; *P* = 0.006) and is given in Figure 5.

MfERG recordings made prior to white PLGA membrane transplantation showed retinal responses in the visual streak to be identical in the left and right eyes and comparable with previously obtained baseline recordings [8] (data not shown). No preimplantation recordings were obtained for the blue membranes. 

## 4. Discussion

In this study, we show that the color of a subretinally transplanted membrane has an effect on the recorded mfERG. We further show a correlation between the brightness of the membrane and the effect on the P1-amplitude ratios in the mfERG. This is to our knowledge the first study that shows correlation of brightness and mfERG. 

The optic disc has no photoreceptors and should therefore not be able to produce an electrical signal when illuminated. Nevertheless, focal illumination of the optic disc is well known to produce an ERG response [11]. This effect has been explained by stray light [11]. Shimada and Horiguchi substantiate this finding in a patient with an optic disc coloboma and show that increased light intensities increase the responses and can even induce a weak full field ERG-response [11]. Photoreceptors adjacent to the disc have been argued to contribute to the recorded response from the optic disc due to limited spatial resolution, eye movements, and forward scatter [12]; Shimada and Horiguchi do not discuss the influence of the color of the coloboma, but the effect of the increased light intensity indicates that color is important, since white surfaces reflect more light than dark surfaces. The membranes implanted in this study have a size of approx 12 mm^2^ and are similar in size to the discussed optic disc coloboma. We show that the P1-amplitude ratio is linearly correlated to the brightness of the membrane, even when we correct for the fundus photo illumination (Figure 5). 

The histological examination showed a low-grade inflammatory degeneration of the outer retina, foreign body giant cell reaction, and formation of choroidal neovascularisation, consistent with the fast degradation of the PLGA membranes with acidic byproducts found by others [13, 14]. We could not demonstrate any histological differences between the white and the blue membranes, and we do not suspect toxicity from the blue dye as it is normally used as an approved food and drug additive [15]. The destruction of the outer retina and complete lack of photoreceptors in all experiments indicated that the membrane-affected areas indeed were without function. We used the highest possible number of hexagons to ensure the highest spatial resolution [16]. This allowed us to use only hexagons from retina completely within the membrane-affected area. The P1-amplitude originates primarily from the bipolar cells and to lesser degree photoreceptors, as has been shown in both pigs [17] and rhesus monkeys [18]. However, with a heavily destroyed outer retina, including photoreceptors, no activation of bipolar cells should take place, and P1-amplitudes should therefore be extinct from within the membrane-affected retinal area [19].

The interindividual variation of the mfERG in the pig is pronounced [8]. Furthermore the mfERG is very susceptible to the depth and length of the anesthesia [20], which accentuates the interindividual and interobservational variation of the mfERG measurements. In addition, the porcine retina is described as having areas of higher cone density within the visual streak [21], which alone could affect the differences found. To overcome the interindividual and interobservational variance, we chose to normalize the data using an identical area in the right, untouched eye. As membrane implanted left eyes were always recorded before the contralateral right control eye, the calculated P1-amplitude ratios of the membrane areas will be too high as it is demonstrated for the corresponding visual streak ratios in Table 2. Both eyes were recorded within a timeframe of 25 minutes and always within the first 2 hours of anesthesia, which should minimize the effect of the anaesthesia on the mfERG-measurements. The difference in mean P1-amplitudes for visual streak between week 0 and 6 combined with the consistency in left/right ratios supports the choice of using the fellow right eye as baseline when evaluating the membrane responses. Regarding the membrane corresponding areas, we found a significant difference in the P1-amplitudes between the control eyes for the blue and white groups. This is most likely due to a higher number of blue implanted membranes centered in the visual streak. Maybe because of the experimental surgical experience gained. Apart from giving higher amplitudes due to a corresponding area completely within the visual streak, the time of anesthesia will also be shorter as it was easier to locate areas near the centered vessels and optic disc prior to mfERG recordings.

In conclusion, brightness and therefore perceived color of a subretinal element influences the P1-amplitude of the mfERG. This should be taken in to account when using mfERG on retinal areas with altered reflective properties. Especially in future retinal tissue engineering studies, mfERG should be used with caution when evaluating local retinal function.

## Figures and Tables

**Table 1:** Distribution of complications after subretinal transplantation of white and blue PLGA membranes.

	Reaction in the vitreous body	Membrane dislocated to the vitreous body	Membrane implanted outside visual streak
White PLGA	2	3	1
Blue PLGA	2	1	1

**Table 2:** Mean P1-amplitude values and ratios of visual streak obtained by multifocal electroretinogram before and 6 weeks after membrane implantation.

	White PLGA Week 0 (*n* = 6)	White PLGA Week 6 (*n* = 6)	Blue PLGA Week 6 (*n* = 5)
Mean P1-amplitude, nV/deg^2^			
SIN visual streak, excl. memb. (SD)	12,08 (3,58)	8,05 (2,18)	11,18 (1,56)
DXT visual streak (SD)	11,37 (4,46)	7,18 (1,74)	9,80 (2,60)
Mean P1-amplitude ratio for visual			
streak (Sin/Dxt) (SD)	1,12 (0,19)	1,12 (0,13)	1,20 (0,28)

SD = standard deviation; Memb. = area of retina with underlying implanted membrane or scarring hereafter; Sin = Left membrane implanted eye; Dxt = right untouched corresponding eye.

**Table 3:** Effect of color of subretinally implanted membranes upon the multifocal electroretinogram 6 weeks after implantation.

	White PLGA (*n* = 6)	Blue PLGA (*n* = 5)	
Mean P1-amplitude, nV/deg^2^			
Memb. (SD)	5,23 (1,70)	5,92 (1,72)	
Memb.-corresp. (SD)	4,75 (1,72)	10,00 (3,72)	
Mean P1-amplitude ratio.			
(Memb./memb.corresp.) (SD)	1,17(0,35)	0,65 (0,26)	(*P* = 0.027)

SD = standard deviation; Memb. = membrane-supported area of retina;

Memb.-corresp. = membrane corresponding area in contralateral control eye.

## Abbreviations

RPC: Retinal progenitor cell
PLGA: Poly lactic-co-glycolic acid
mfERG: Multifocal electroretinogram
ARVO: The Association for Research in Vision and Ophthalmology
i.v.: Intravenous
IR: Infrared
HE: Hematoxylin and eosin
wt%: Weight percent
ONL: Outer nuclear layer
INL: Inner nuclear layer.
